# Your Unconscious Knows Your Name

**DOI:** 10.1371/journal.pone.0032402

**Published:** 2012-03-05

**Authors:** Roland Pfister, Carsten Pohl, Andrea Kiesel, Wilfried Kunde

**Affiliations:** Department of Psychology, Julius-Maximilians-University of Würzburg, Würzburg, Germany; Royal Holloway, University of London, United Kingdom

## Abstract

One's own name constitutes a unique part of conscious awareness – but does this also hold true for unconscious processing? The present study shows that the own name has the power to bias a person's actions unconsciously even in conditions that render any other name ineffective. Participants judged whether a letter string on the screen was a name or a non-word while this target stimulus was preceded by a masked prime stimulus. Crucially, the participant's own name was among these prime stimuli and facilitated reactions to following name targets whereas the name of another, yoked participant did not. Signal detection results confirmed that participants were not aware of any of the prime stimuli, including their own name. These results extend traditional findings on “breakthrough” phenomena of personally relevant stimuli to the domain of unconscious processing. Thus, the brain seems to possess adroit mechanisms to identify and process such stimuli even in the absence of conscious awareness.

## Introduction

The own name is among the first concepts that a human being encounters. It is experienced countless times throughout the entire lifespan and it is one of the most resilient entries in memory. The sound of one's spoken name and the appearance of its written equivalent therefore constitute a unique part of our conscious experience. Accordingly, there is good evidence that the own name is processed in a privileged manner. Most notably, the own name has the power to reach awareness in conditions in which most other stimuli remain unnoticed: the well-known “breakthrough” phenomenon [1].

Whereas privileged access to consciousness is one peculiarity of the own name, little is known about such peculiarities in situations in which the own name does not reach awareness. In other words, it is unknown, whether the own name is also unique for unconscious processing. The present study tests the hypothesis that the own name is processed in a privileged manner despite remaining unaware. Support for this hypothesis comes from recent studies on patients with disorders of consciousness such as patients in the vegetative state or the minimally conscious state [2]–[3]. These patients were confronted with their own names as oddballs among other auditory stimuli, and the own name elicited a pronounced mismatch negativity in the EEG signal (see also [4]–[5]). In fact, a distinctive electrophysiological response to the patient's own name was observed at least in a subset of the patients, and the presence of this response predicted subsequent recovery. Thus, the own name seems to be processed even with diminished consciousness – indicating that it might have a special role for entirely unconscious processing.

In the current study we examined under more controlled conditions whether the own name plays a unique role for unconscious processing in healthy individuals. To this end, we employed the subliminal priming paradigm, a well-established method to explore the impact of unconsciously presented stimuli [6]–[8]. In this paradigm, participants respond to a target which is preceded by a subliminal prime stimulus. The prime occurs very briefly and it is masked to prevent it from entering conscious awareness. Yet, even though the prime is rendered invisible, it nevertheless influences the response to the following target because the prime already (pre)activates the associated response. Consequently, responding is faster if prime and target call for the same response as compared to different responses, indicating unconscious prime processing.

Subliminal stimuli are, however, not processed inevitably. More precisely, whereas primes that are also presented as targets normally give rise to strong and robust priming effects across various conditions [9], *novel primes* that are exclusively presented as primes throughout the experiment are only effective under certain conditions (for recent reviews see [10]–[12]).

One condition that renders such novel primes effective is when the prime stimuli are expected. Manipulating participants' expectations about potentially occurring stimuli is possible by using either small or large target sets, e.g., by employing either 4 or 40 different stimuli as targets for two to-be-discriminated categories [13]–[14]. With large target sets, novel primes typically elicit significant priming effects whereas with small target sets, they do not (cf. also [9], [15]–[17]). With large target sets, participants likely expect many different exemplars from the same semantic category as the experienced targets. In contrast, small target sets give rise to circumscribed expectations about the specific target stimuli, e.g., their specific perceptual appearance [14], [16]. Consequently, a novel prime will be expected in the former context but not in the latter context and only primes that are expected in the current context tend to elicit unconscious priming effects (for similar results, see [18]–[20]).

**Figure 1:**
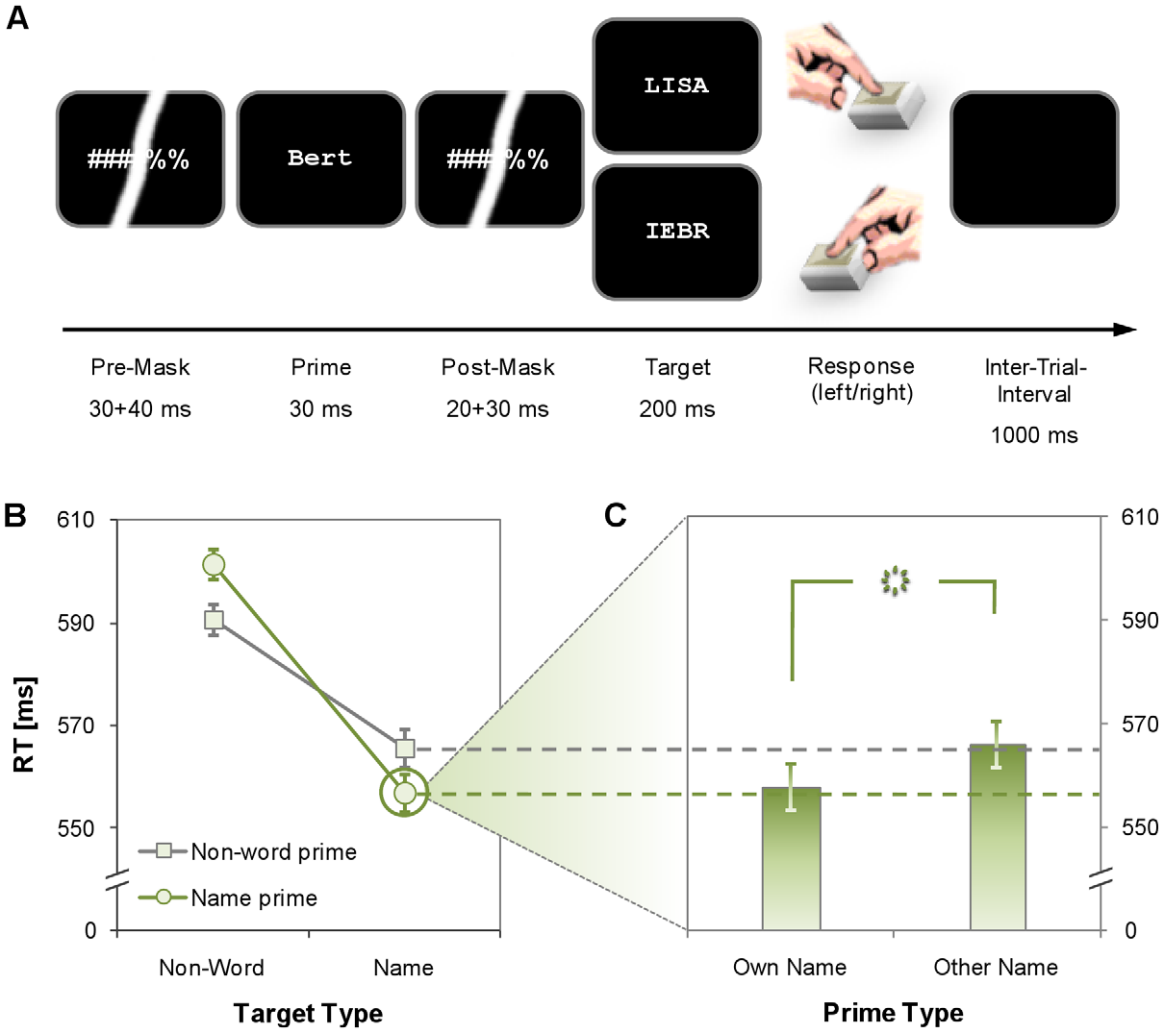
Design and results of the experiment. (**A**) Participants classified a target as name or non-word; the target was always preceded by a masked unconscious prime stimulus. Unbeknown to the participants, among these primes were their own name and the name of another, yoked participant. (**B**) Participants responded significantly faster when prime and target called for the same response than for opposite responses, revealing typical priming effects. Error-bars indicate 95% within-subjects confidence intervals, computed separately for each target type. (**C**) A more detailed analysis of name targets revealed that the own name facilitated responding similar to target primes whereas the other name did not. Error-bars indicate 95% within-subjects confidence intervals for the difference between own and other name primes.

The present experiment employs such a small target set to explore whether the own name as an exceptionally important concept is also subject to this general restriction or whether the own name can be processed unconsciously even in the absence of explicit expectations. The latter hypothesis is in line with recent studies showing neurophysiological responses to personal significance as early as 40 ms after stimulus onset [21], i.e., on a timescale that might be suitable to influence unconscious processing [22]–[23]. To test this hypothesis, our participants decided as fast as possible whether a target stimulus was a name or a non-word (see Figure 1A). Targets were two female surnames, two male surnames, and four meaningless letter strings. The preceding subliminal prime was either a potential target stimulus that participants practised during the experiment or a stimulus that never appeared as target. Unbeknown to the participants, their *own name* was among these novel primes as well as the name of another, yoked participant (*other name*).

Crucially, we used a rather small target set of only four target names and letter strings, i.e., we deliberately set up conditions that render typical novel primes ineffective, even when these primes are in close semantic relation to the following target [13]. Accordingly, we expected the other name not to facilitate responses to name targets. The own name, by contrast, might still be processed unconsciously which would result in a priming effect for the own name even though other unexpected primes are rendered ineffective.

## Methods

### Participants

Twenty-four right-handed participants (12 males; mean age: 22.9 years) were tested in individual sessions and were assigned to yoked pairs of one male and one female participant each. The yoking procedure ensured that both participants of each pair encountered exactly the same stimuli throughout the experiment even though the stimuli differed in personal significance (i.e., the own name stimulus of participant 1 would be the other name stimulus for participant 2, and vice versa). Consequently, a differential impact of own and other name could not be due to genuinely different perceptual relations of these novel primes to the target stimuli. All participants reported normal or corrected-to-normal vision and were naive concerning the hypothesis of the present study. Oral consent was acquired from each participant prior to the experimental session and participants were debriefed regarding the yoking procedure at the end of the session. Because this was a non-clinical study without any harming procedure, formal ethical approval was not sought. The data were analyzed anonymously and we can ensure that participants' identities are protected.

### Design and Procedure

Participants decided as fast as possible whether a letter string on the computer screen was a name or a non-word by pressing a left or a right response key (viewing distance: ca. 60 cm; monitor frequency: 100 Hz; all stimuli appeared in 36 pt Courier New font). Target stimuli were four German names (“RAINER”, “RENATE”, “LISA”, “BERT”) and four non-words (“ARSBNR”, “SRBRAR”, “IEBR”, “AIST”) and the target-key mapping was counterbalanced across participants. The target was preceded by a masked prime stimulus that was either among the potential target stimuli, one of two additional non-words (“Neti”, “Irtaes”), or, crucially, the participant's own name or the name of his or her yoked partner. Targets always appeared in capital letters while primes were spelled naturally with only the first letter capitalized. The prime was embedded into pre- and post-mask, both of which consisted of a rapid succession of 8 hash signs (“########”) and the same number of percent signs (“%%%%%%%%”).

More precisely, each trial began with the pre-mask (30 ms hash signs and 40 ms percent signs) followed by prime (30 ms) and post-mask (20 ms hash signs and 30 ms percent signs). Then, the target appeared for 200 ms. Finally, the screen was blanked while the program waited up to 2000 ms for a response. Errors and response omissions triggered a 1000 ms error message (German words “Fehler!” for errors, “Bitte schneller!” for omissions) whereas the screen remained blank for the same period after correct responses. The next trial started after an inter-trial-interval of 1000 ms.

Participants worked through 5 blocks of 128 trials each (8 target primes×8 targets+4 novel primes×8 targets×2). Afterwards, participants were debriefed and completed an additional signal detection task to test for prime visibility. The signal detection task was similar to the main experiment but participants were asked to make unspeeded responses to the prime instead of the target. This task comprised a single block of 128 trials. The results of the signal detection task confirmed that the primes and especially the own name were indeed unconscious; d' = −0.04, *t*(23) = −1.28, *p* = .214 for all stimuli; d' = 0.03, *t*(23) = 0.41, *p* = .682 for the own name. These results suggest that the present setup effectively prevented a breakthrough of the own name and allows us to investigate how the own name is processed unconsciously.

## Results

Response times were analyzed in two separate steps. First, we ran a 2×2 repeated-measures ANOVA with the factors of prime type (name vs. non-word) and target type (name vs. non-word) on the entire data set to validate the employed priming paradigm. Then, we analyzed the priming effect for name targets more thoroughly by systematically comparing the different prime types. For all reaction time (RT) analyses, we excluded error trials (7.3%) and outliers deviating more than 2.5 standard deviations from the mean of the design cell (calculated separately for each participant; <2.2% for all analyses).

Figure 1B shows that responses were facilitated when prime and target called for the same response than for opposite responses, revealing typical congruency effects. That is, participants responded faster when prime and target were both names or both non-words as compared to the reverse combinations, *F*(1, 23) = 28.35, *p*<.001, η_p_^2^ = .55. Additionally, responses were faster for name targets than for non-word targets, *F*(1, 23) = 12.89, *p* = .002, η_p_^2^ = .36, whereas the main effect of prime type did not approach significance (*F*<1).

A similar analysis of the error data confirmed that the crucial interaction in the RT data did not result from a speed-accuracy trade-off. In fact, the interaction was also significant for error percentages, *F*(1, 23) = 14.18, *p* = .001, η_p_^2^ = 0.38. Participants committed fewer errors when a name target was preceded by a name prime (5.9%) than by a non-word prime (8.3%). Conversely, non-word targets gave rise to more errors when preceded by name primes (8.0%) as compared to non-word primes (6.8%). The main effects of target type (*F*<1) and prime type were not significant, *F*(1, 23) = 2.30, *p* = .143, η_p_^2^ = 0.09.

Based on these effects, we further analyzed the differential contributions of different primes to the congruency effect for name targets. Crucially, the own name facilitated responding to a name target to the same extent as a name that participants practiced as visible target dozens of times, *t*(23) = 1.36, *p* = .187 (Figure 1C). To the contrary, the other name had the same effect as presenting a non-word prime, *t*(23) = 0.14, *p* = .887, a typical observation for unexpected stimuli when the number of different targets is as small as in the present study [14], [19]. Consequently, participants responded faster when the own name preceded a target name as compared to the other name, *t*(23) = 2.68, *p* = .013. Thus, the own name primes a name target, whereas another unexpected name does not. To corroborate this conclusion, we further dissected the congruent name target primes into identical (e.g. “Lisa”>“LISA”) and non-identical (e.g. “Lisa”>“BERT”) target primes. In line with previous studies (e.g., [7], [24]), identical target primes had a tremendous effect and gave rise to the fastest responses (540 ms), clearly exceeding the effect of non-identical target primes (556 ms), *t*(23) = 4.85, *p*<.001, as well as the effect of the own name (558 ms), *t*(23) = 3.71, *p* = .001. This additional analysis, however, also showed a remarkably similar effect of the own name and non-identical target primes, *t*(23) = 0.41, *p* = .683.

As for the RT data, the differential analysis of error percentages (for name targets) also yielded a slight advantage of identical target primes (3.8%) as compared to the other three prime types combined, *t*(23) = 2.81, *p* = .010. The error percentages for non-identical target primes (5.8%), the own name (7.2%), and the other name (6.3%), however, did not differ at all, *p*>.134 for each pairwise comparison. As for the first step of the analysis, these results effectively rule out a speed-accuracy trade-off and allow for a straightforward interpretation of the RT data. In addition, it should be noted that we deliberately restricted this differential analysis to name targets because the identification of non-words is typically assumed to rely on qualitatively different processes than the identification of words (see [24], for an extended discussion in the context of the repetition priming paradigm). Consequently, the impact of own and other name did not differ with regard to non-word targets, *t*(23) = 0.90, *p* = .379, and both gave rise to reliably faster reactions than potential target names did (*p*s<.002).

## Discussion

The present study set out to investigate whether one of the most important concepts for any individual – the own name – is preferentially processed unconsciously. To this end, we employed the subliminal priming paradigm and presented the participant's own name as an unexpected prime stimulus before name and non-word targets. The name of another, yoked participant served as a control stimulus. Crucially, we used a small target set, a condition that normally renders such novel primes ineffective [13]. This was indeed the case for the other name which did not facilitate responses to subsequent target names. The own name, however, showed such a priming effect, indicating that it was processed unconsciously. Thus, unconscious processes do not seem to discriminate mandatorily between a non-word and a novel name whereas the own name is readily processed even without reaching conscious awareness.

This pattern of results departs from classic psychological findings on the astonishing potential of the own name to capture attention and its preferential access to consciousness (the well-known “breakthrough” phenomenon; [1], [25]–[30]). Such a breakthrough would have been evident in an increased visibility of the own masked name. The own name, however, was just as undetectable as any other stimulus what is evident from the present signal detection results. Thus, we demonstrate that the own name is processed preferentially, even if its neural representation is too weak to reach consciousness eventually [31].

We conjecture that the own name belongs to a limited set of personally relevant stimuli that are obligatorily processed up to a semantic level of analysis even in the absence of conscious awareness [21], [32]. Support for this conclusion comes from recent electrophysiological studies that showed marked responses to one's own name in conditions of reduced consciousness such as sleep [33] and the minimally conscious state [2]–[4]. Such preferential unconscious processing of the own name has intriguing implications for current theories of consciousness such as Global Workspace Theory [31]. Modern computational versions of this theory assume consciousness to arise from biased competition of separate modular processes (e.g., [34]). The present results suggest that specific stimuli such as the own name can be processed very elaborately even if these processes are not in the current focus of the cognitive workspace and do therefore not gain access to consciousness.

But what exactly is the mechanism behind this preferential unconscious processing? Whereas several factors, such as personal significance [25], [27], emotional valence [35], and attention [36], [37] were shown to influence the conscious identification of (personally relevant) stimuli, it is not clear whether these factors influence unconscious processing in a similar way. Consequently, the preferential unconscious processing of these stimuli might rely on factors such as personal significance or, importantly, the enormous experience with this particular stimulus. Alternatively, the own name might not be obligatorily processed semantically but instead it might be more efficient than other names in attracting attention in the absence of conscious awareness, which might also foster its processing [38]. Disentangling these processes certainly deserves further elaboration. In any case, the present results clearly show that humans are not only eager to detect their own name as readily as possible – but that unconscious processes can identify the own name already beforehand and possibly even without telling. In other words: Your unconscious knows your name.
